# Supplementary material for: AGO-bound mature miRNAs are oligouridylated by TUTs and subsequently degraded by DIS3L2
Source: Nat Commun. 2020 Jun 2;11:2765. doi: 10.1038/s41467-020-16533-w (PMC7265490; doi:10.1038/s41467-020-16533-w)
Supplement: Supplementary file 1 — Supplementary Information [file 41467_2020_16533_MOESM1_ESM.pdf]

Supplementary Information

AGO-bound Mature miRNAs are Oligouridylated by TUTs and Subsequently Degraded by DIS3L2

Yang et al.

Supplementary Figures 1-6
Supplementary Table 1

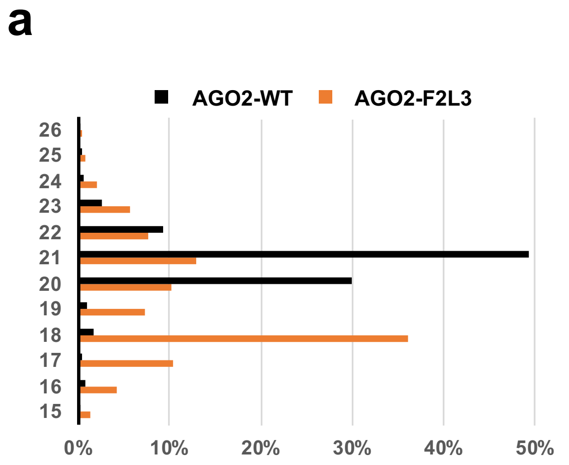

b

Top 10 reads of AGO2-F2L3-IP

Sequence	Length (nt)	Relative abundance
UUCACAGUGGCUAAGUUC	18	31.3%
UUCACAGUGGCUAAGUU	17	8.4%
UUCACAGUGGCUAAGUUCCG	20	7.0%
UUCACAGUGGCUAAGUUCCGA	21	5.5%
UUCACAGUGGCUAAGUUCCGU	21	4.6%
UUCACAGUGGCUAAGU	16	3.5%
UUCACAGUGGCUAAGUUCU	19	3.2%
UUCACAGUGGCUAAGUUU	18	2.2%
UUCACAGUGGCUAAGUUCCGUU	22	2.1%
UUCACAGUGGCUAAGUUCC	19	2.0%

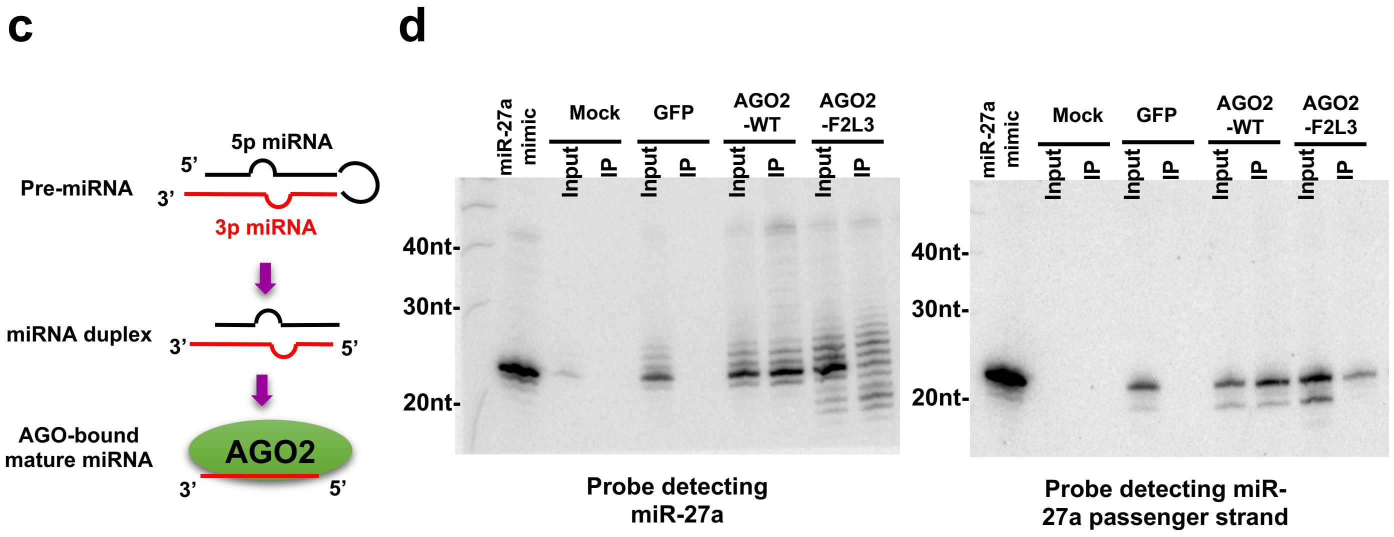

Supplementary Fig. 1 | Disrupting the binding between the AGO PAZ domain and miRNA 3' end promotes miRNA 3' modifications.
a, Comparing the length distribution of AGO2-WT with AGO2-F2L3 bound miRNA reads. **b,** The sequences of the top 10 abundant AGO2-F2L3 bound miRNA-27a-3p reads. **c,** Illustration of miRNA processing. **d,** Co-expression of synthetic miR-27a siRNA mimic with GFP or Flag-Ago2 constructs: Mock (untransfected), wild-type (WT), and PAZ mutant (F2L3). Detection of the guide and passenger strand of miR-27a in the input and Flag-immunoprecipitate (IP) by Northern blot. Source data are provided as a Source Data file.

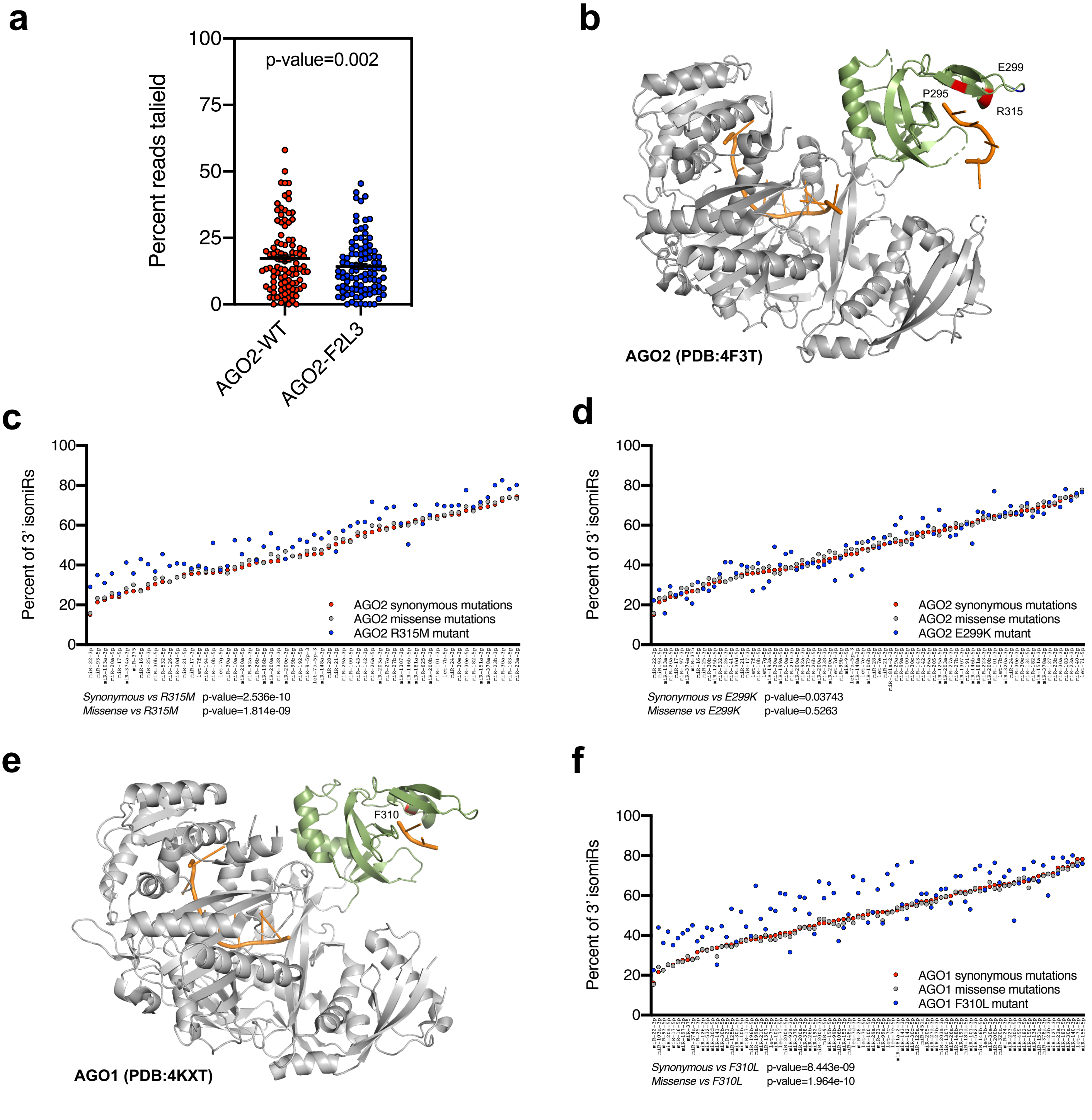

Supplementary Fig. 2 | Endogenous miRNAs with an exposed 3' end are subjected to extensive 3' modifications.

a, Plot of percentage of tailed reads in endogenous miRNA. (Mean ± SEM, N=100 miRNAs). **b,** Structure of AGO2 (PDB:4F3T) with mutations in the PAZ domain correlating with increased 3' isomiRs (red) or neutral (blue) in TCGA patients. **c,d,** Plot of percentage of isomiRs for the endogenous miRNA with highest expression in TCGA samples with synonymous (51 patients), missense (81 patients), R315M and E299K mutations on AGO2. Wilcoxon's test for paired values were used to calculate the p-values. **e,** Structure of AGO1 (PDB:4KXT) with mutations in the PAZ domain correlating with increased 3' isomiRs (red) in TCGA patients. **f,** Plot of percentage of isomiRs for the endogenous miRNA with highest expression in TCGA samples with synonymous (25 patients), missense (73 patients) and F310L mutations on AGO1. Wilcoxon's test for paired values (two-sided) were used to calculate the p-values. Source data are provided as a Source Data file.

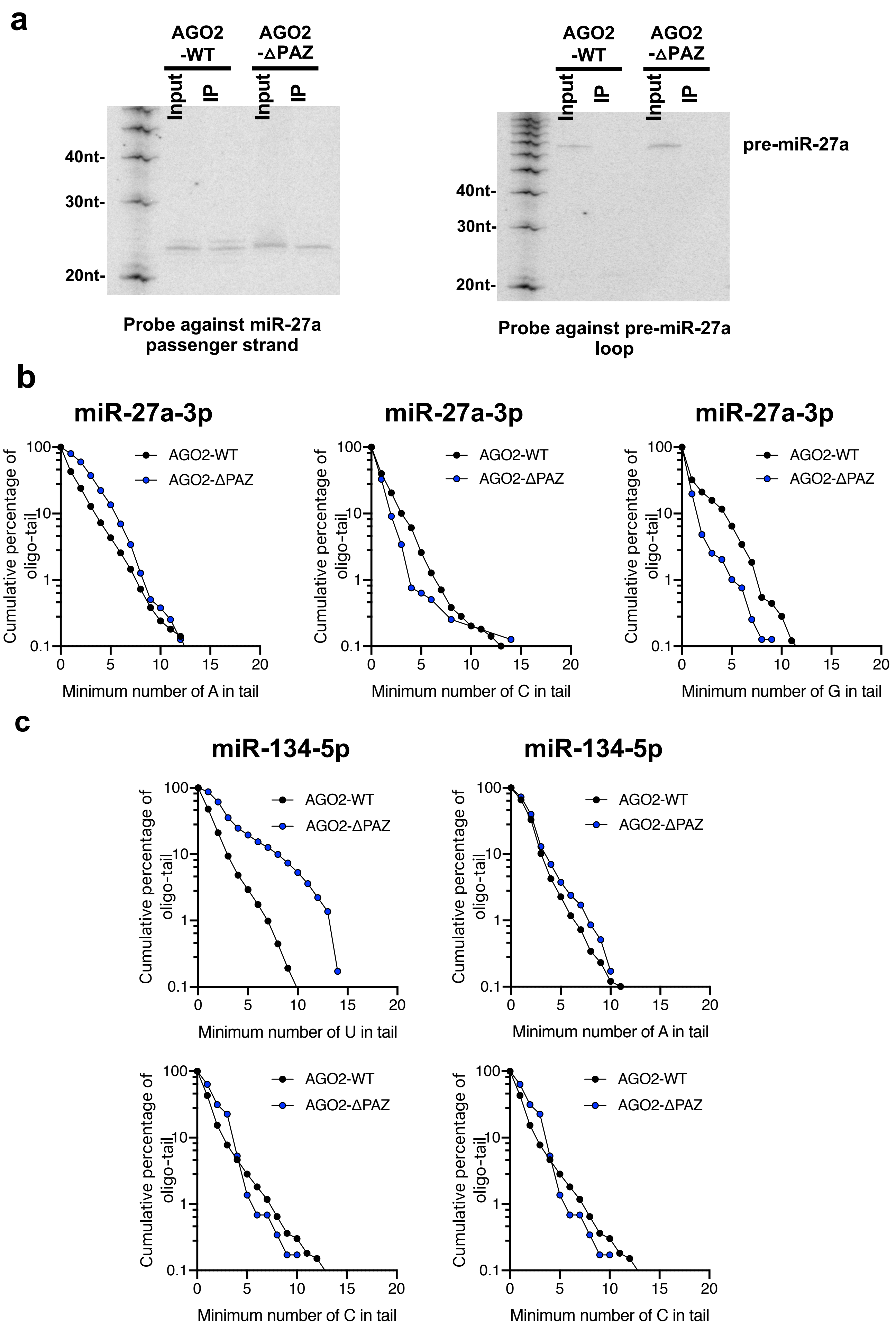

Supplementary Figure 3

d

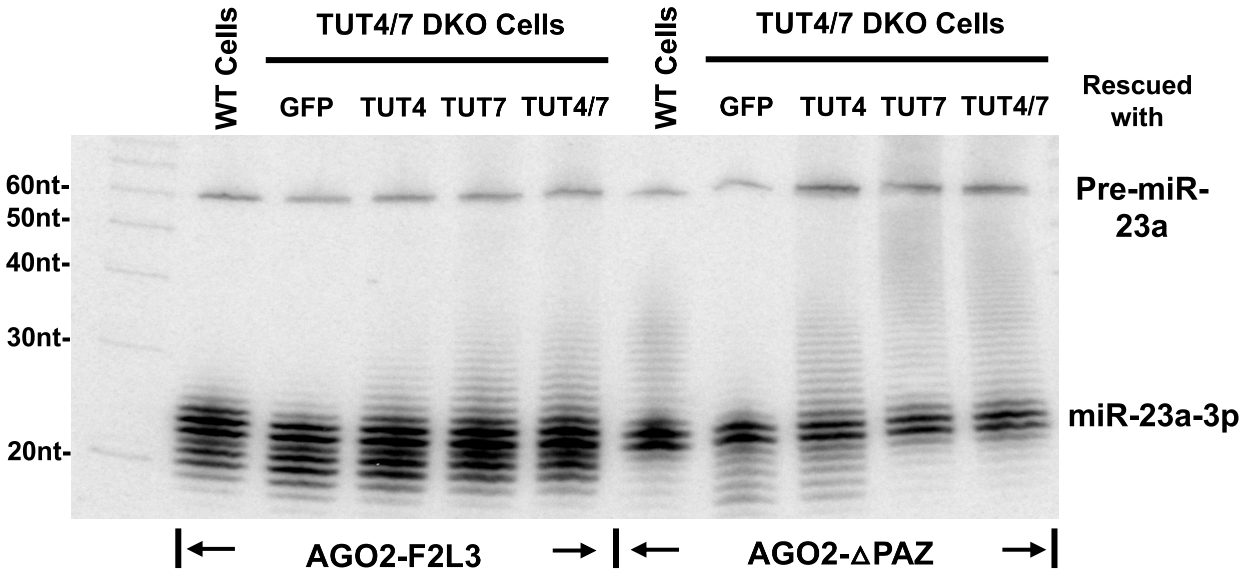

Supplementary Fig. 3 | Ago-bound miRNAs with an exposed 3' end are oligo-uridylated by TUT4 and TUT7.
a, Co-expression of pri-miR-27a and FLAG-AGO2 constructs: wild-type (WT) and PAZ domain deletion (ΔPAZ). Probes against the passenger strand and loop of miR-27a were used to detect miR-27a-5p and pre-miR-27a in the input and Flag-immunoprecipitate (IP). **b,** Nucleotide composition of miR-27a-3p oligo-tails. Horizontal axis indicates the absolute number of non-templated A, C, or G nucleotides in the tails, and the vertical axis indicates the cumulative percentage from the longest to the shortest oligo-tail. **c,** Nucleotide composition of miR-134-5p oligo-tails bound to AGO2-WT and AGO2-ΔPAZ. Horizontal axis indicates the absolute number of non-templated U, A, C, and G nucleotides respectively in the tails, and the vertical axis indicates the cumulative percentage from the longest to the shortest oligo-tail. **d,** Co-expression of pri-miR-27a and FLAG-AGO2 constructs: Wild-type (WT) and PAZ domain deletion (ΔPAZ). Detection of miR-27a-3p in input and Flag-immunoprecipitate (IP) by Northern blot. Source data are provided as a Source Data file.

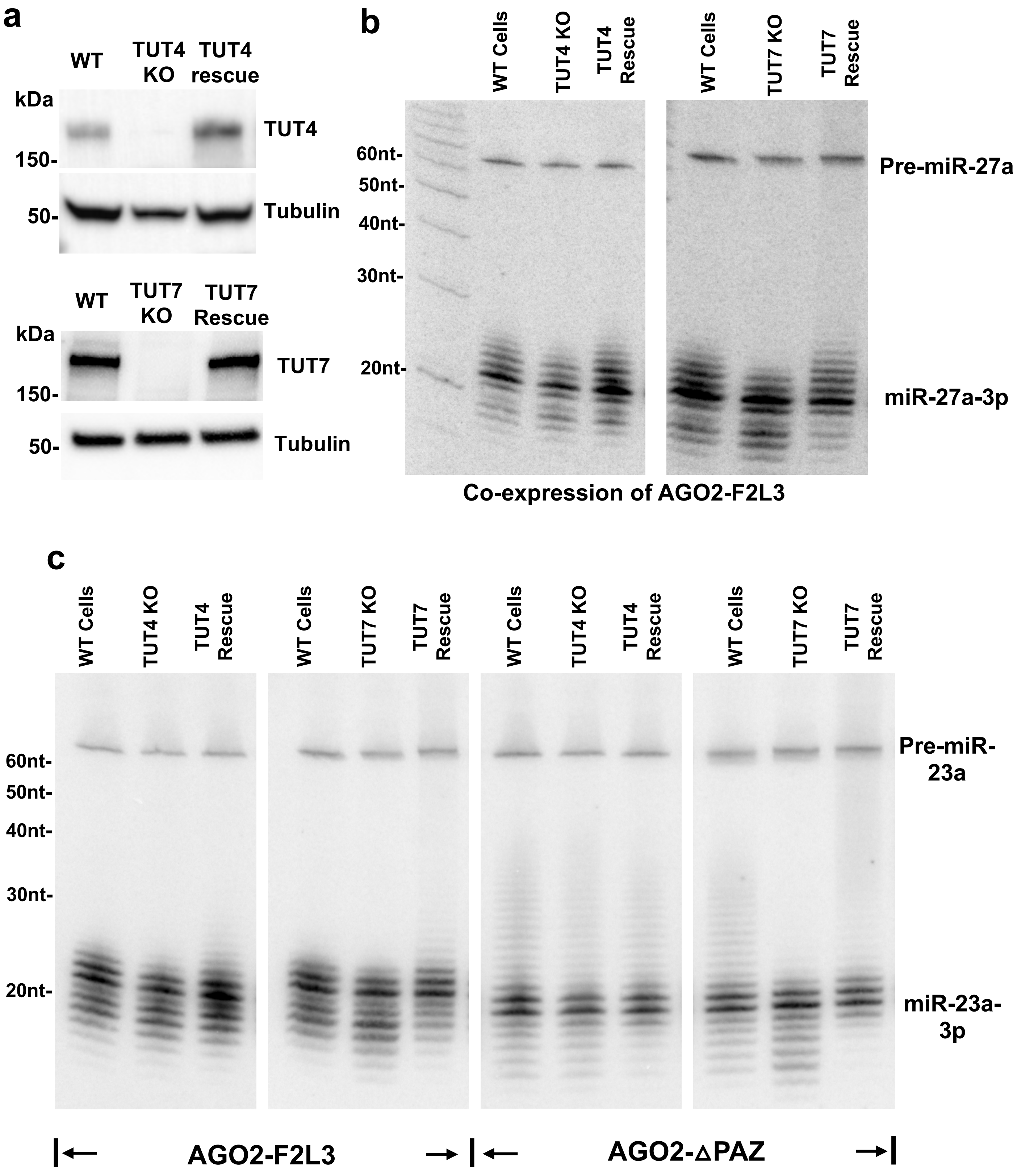

Supplementary Fig. 4 | TUT7 is robust in oligo-uridylating mature miRNAs due to its association with the Argonaute proteins.
a, Western blot detecting protein levels of TUT4 and TUT7 in single knockout cell lines with or without rescue. **b,** Detection of miR-27a-3p in Wild type, TUT4 KO and TUT7 KO cells co-expression of pri-miR-27a and AGO2-F2L3 with or without TUT rescue by Northern blot. **c,** Detection of miR-23a-3p in Wild type, TUT4 KO and TUT7 KO cells transfected with pri-miR-23a and AGO2-F2L3 or AGO2-ΔPAZ with or without TUT rescue. Source data are provided as a Source Data file.

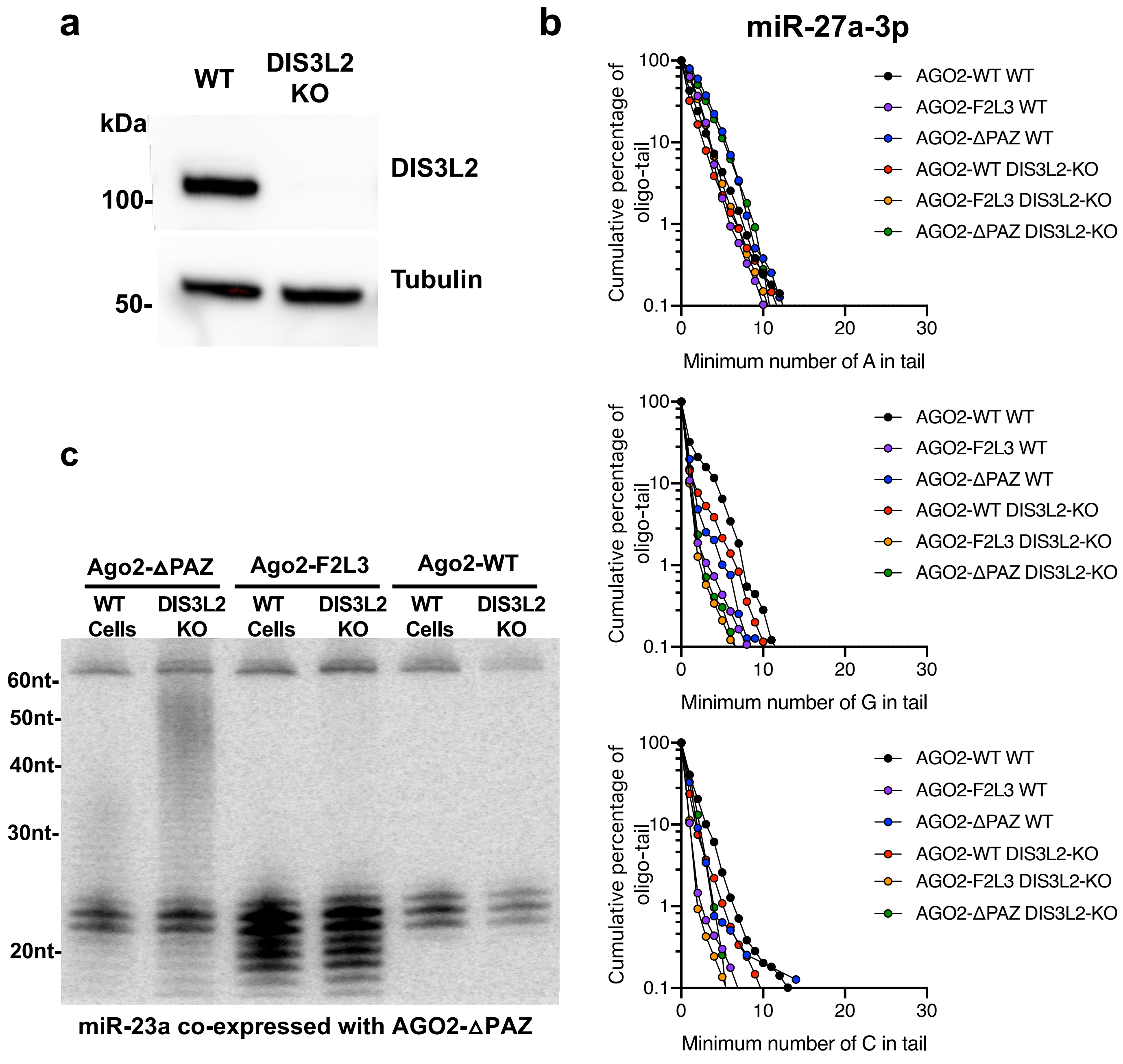

Supplementary Figure 5

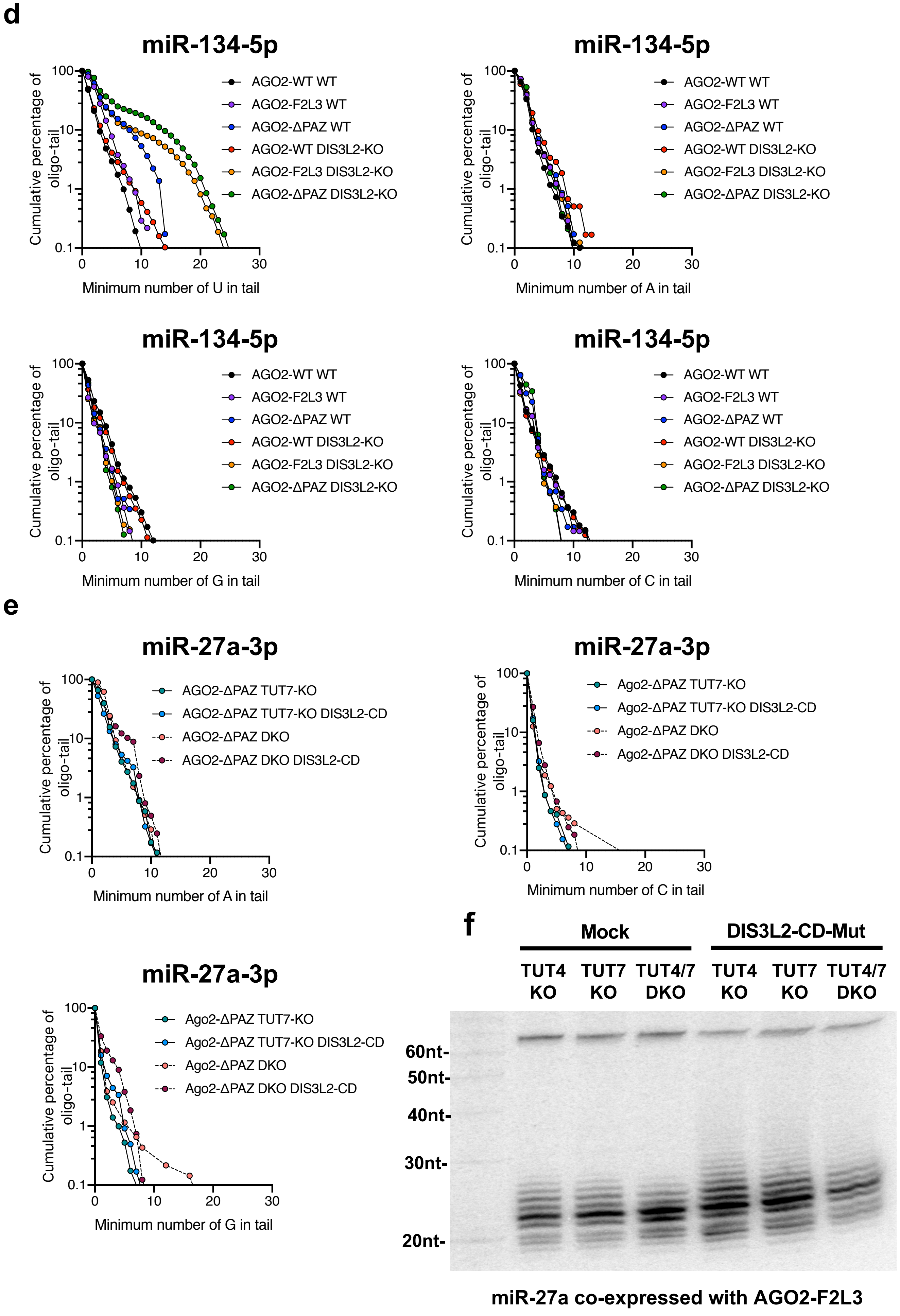

Supplementary Figure 5

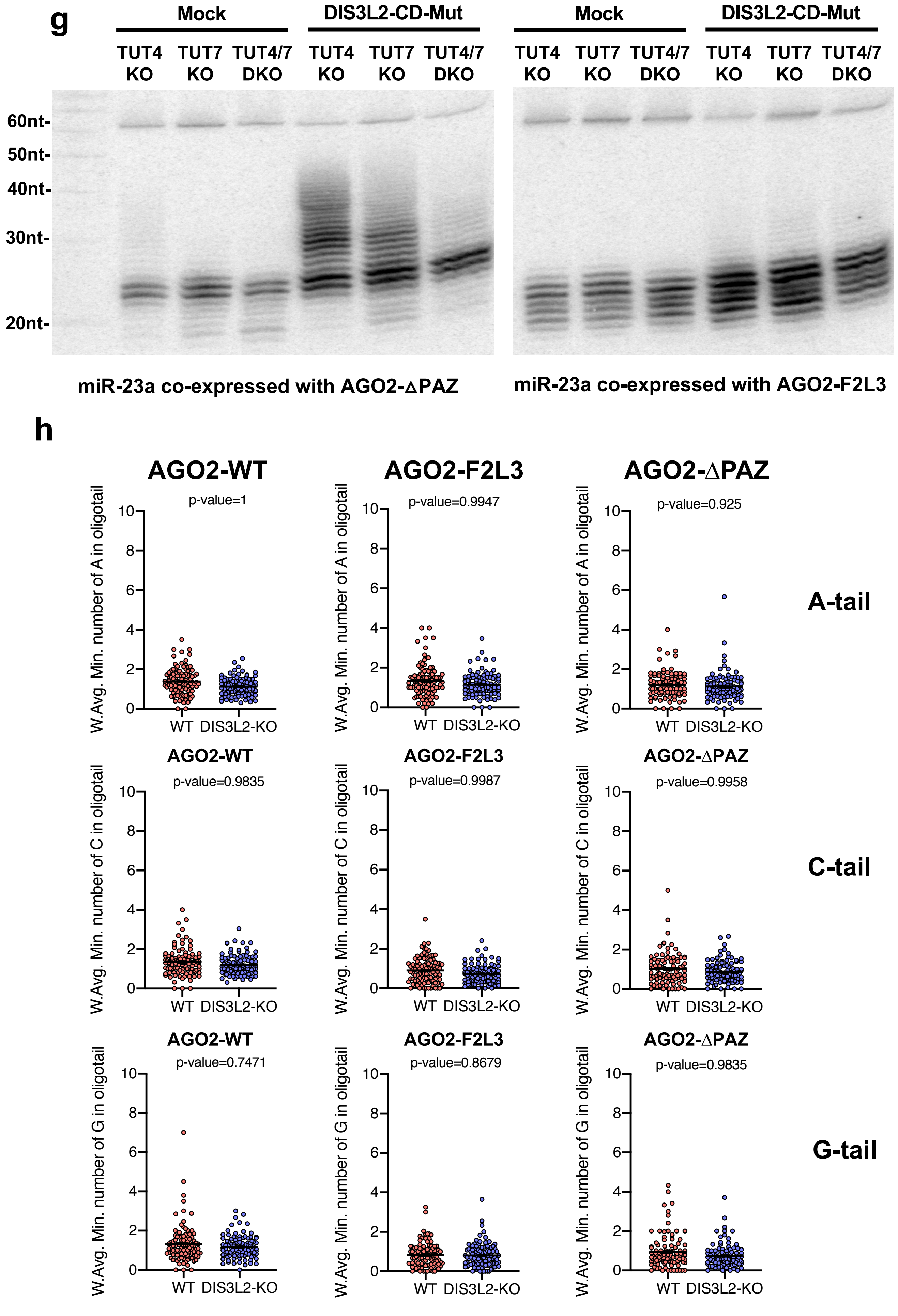

Supplementary Figure 5

i

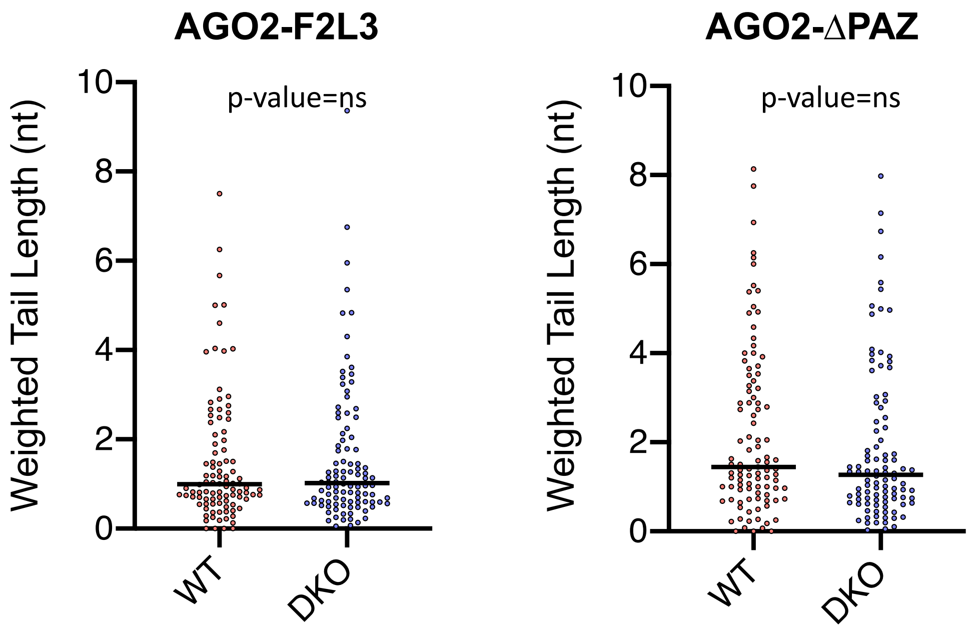

j

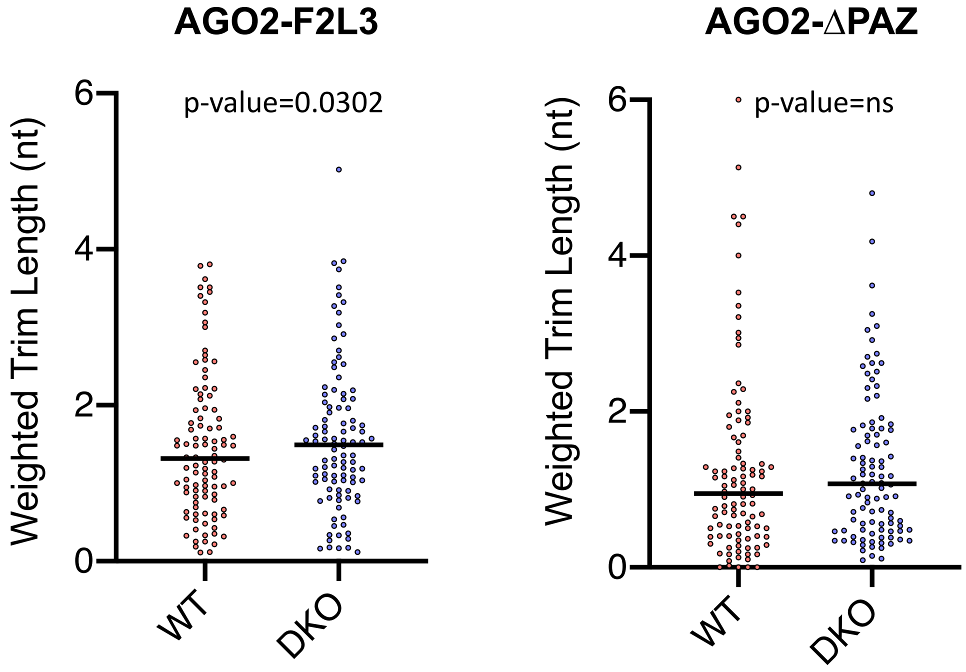

Supplementary Figure 5

Supplementary Fig. 5 | DIS3L2 degrades oligo-uridylated mature miRNAs.
a, DIS3L2 KO cell line is confirmed by Western blot detecting the protein level of DIS3L2. **b,** Composition of miR-27a-3p oligo-tail, number of A, C, or G nucleotides in the tail (x-axis) and cumulative percentage (y-axis), comparing Ago2 constructs (WT, F2L3 and ΔPAZ) in wild-type and DIS3L2 KO backgrounds. **c,** Co-expression of pri-miR-23a and Flag-AGO2 constructs (WT, F2L3 and ΔPAZ) in HEK293T wild-type and DIS3L2 KO background. Detection of miR-23a-3p in input and Flag-immunoprecipitate (IP) by Northern blot. **d,** Composition of miR-134-5p oligo-tail, number of U, A, C, or G nucleotides in the tail (x-axis) and cumulative percentage (y-axis), comparing AGO2 constructs (WT, F2L3 and ΔPAZ) in wild-type and DIS3L2 KO backgrounds. **e,** Composition of miR-27a-3p oligo-tail, number of A, C, and G nucleotides in tail (x-axis) and cumulative percentage (y-axis), comparing DIS3L2-CD-Mut U-tail protection in TUT7 KO and TUT4/7 DKO backgrounds. **f,g,** Detecting miR-27a-3p (**f**), miR-23a-3p (**g**) in the single and double TUT4 and TUT7 KO cells co-expression of pri-miR-27a cluster, with DIS3L2 catalytic-dead mutant (DIS3L2-CD-Mut) and AGO2-F2L3 (**f,g**) or AGO2-ΔPAZ (**g**) by Northern blot. **h,** Weighted average number of oligo A, C or G tail nucleotides added to endogenous miRNA loaded into AGO2 (WT, F2L2 and ΔPAZ) in wild-type and DIS3L2 KO backgrounds. **i,j,** Weighted tail length (**i**) and trim length (**j**) in endogenous miRNA bound to AGO2-F2L3 and AGO2-ΔPAZ. Wilcoxon's test for paired values (two-sided) were used to calculate the p-values. Source data are provided as a Source Data file.

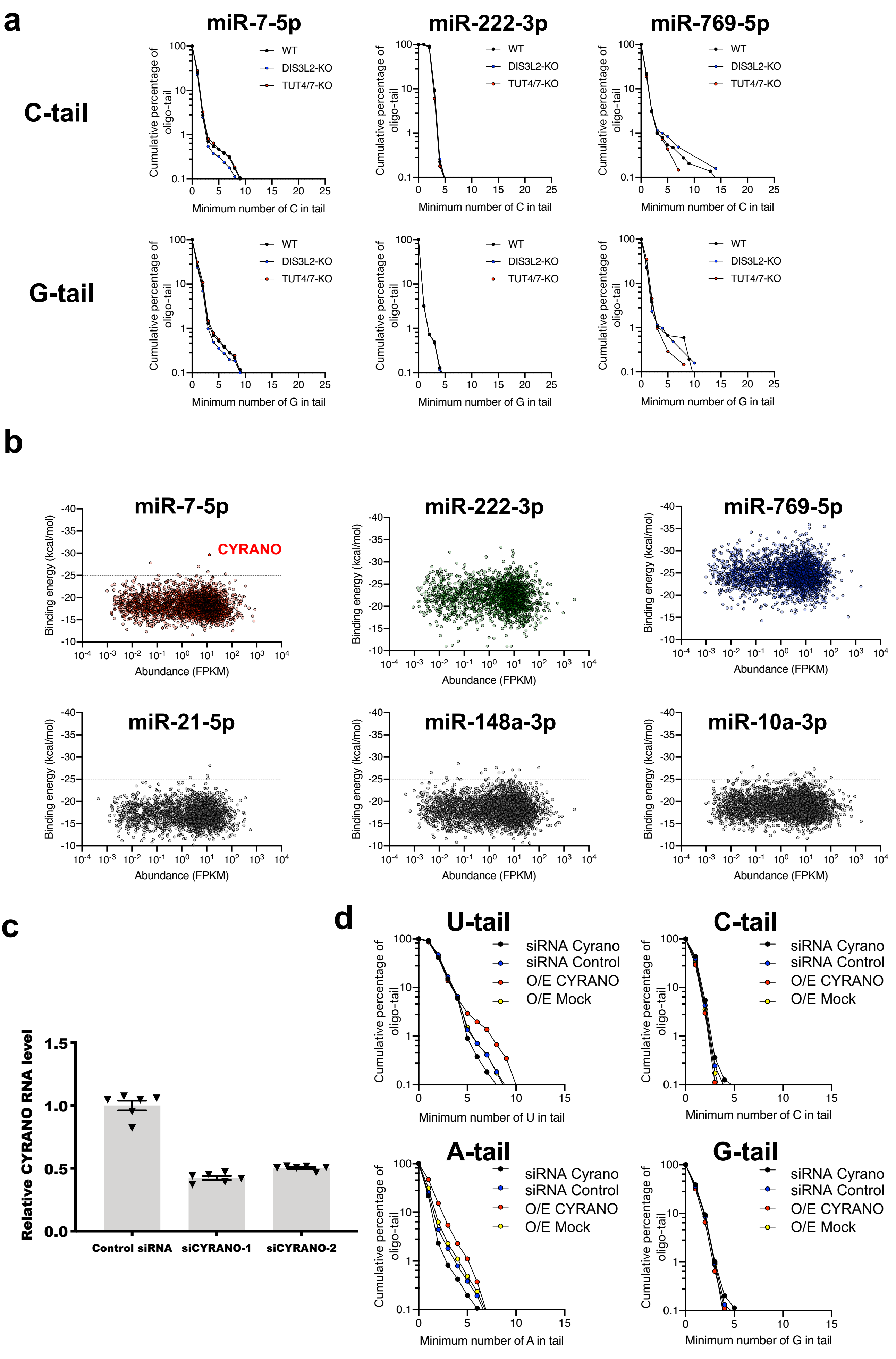

Supplementary Figure 6

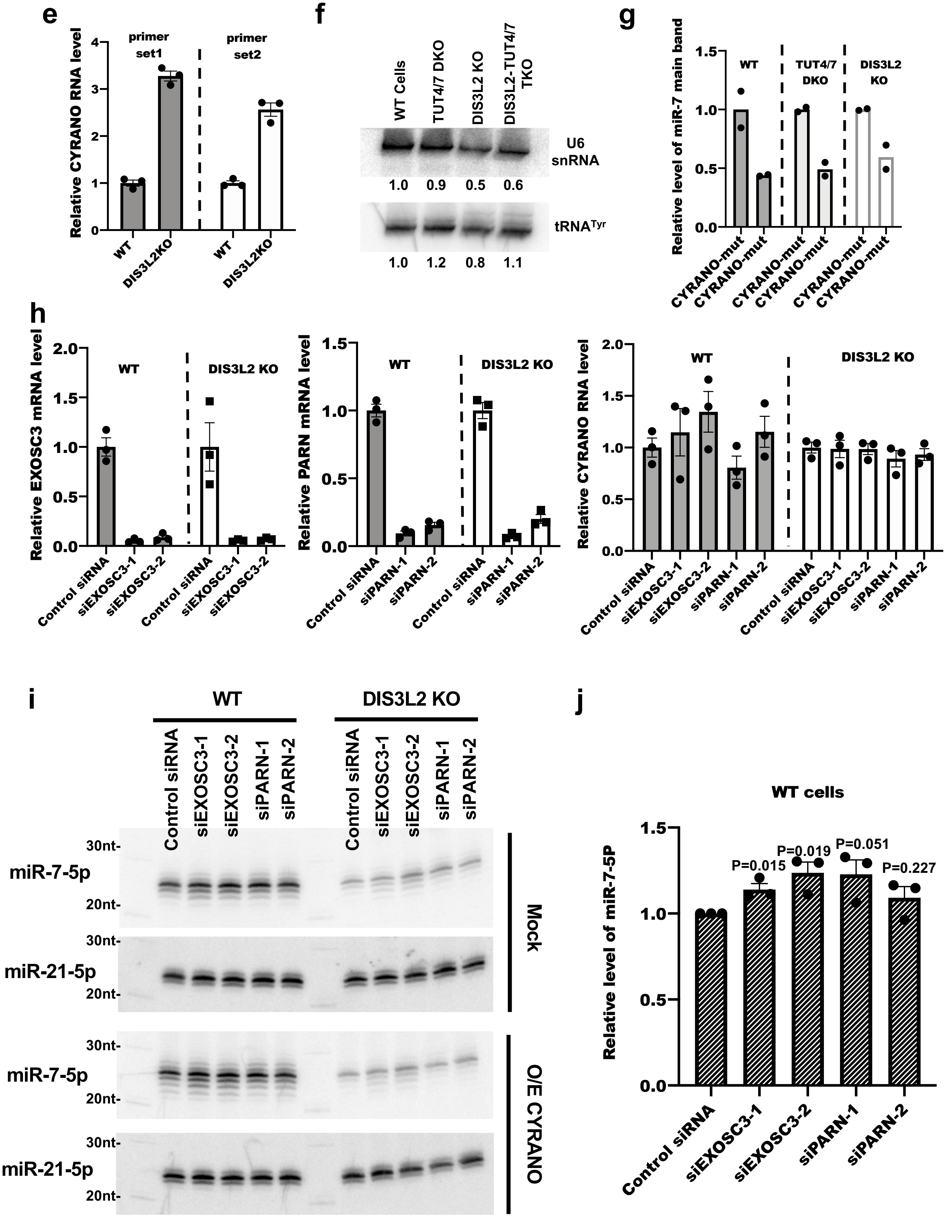

Supplementary Figure 6

Supplementary Fig. 6 | TUT-DIS3L2-mediated decay is implicated in but not required for TDMD.

a, Comparing the composition of oligo-tail (number of C or G in the tail, x-axis) and cumulative percentage (y-axis) of miR-7-5p, miR-222-3p and miR-769-5p, in wild-type, TUT4/7 DKO and DIS3L2 KO cells. **b,** Scatter plot on target RNA abundance (FPKM) and binding energy (kcal/mol) of the miRNA:target duplex. **c,** Real-time qPCR confirmed the knockdown efficiency of CYRANO by two siRNA sequences. **d,** Comparison of the composition of oligo-tail (number of U, A, C or G in the tail, x-axis) and cumulative percentage (y-axis) of miR-7-5p when knockdown or over-expression CYRANO RNA in wild-type HEK193T cells. **e,** Real-time qPCR comparing the Cyrano RNA level in the wild-type and DIS3L2 KO cells. **f,** Northern blot of U6 snRNA and Tyr-tRNA in wild-type, TUT4/7 DKO, DIS3L2 KO and DIS3L2-TUT4/7 TKO cells. **g,** Quantitating the density of the miR-7-5p main band shown in the Fig. 6e. **h,** Real-time qPCR confirmed the knockdown efficiency of EXOSC3 and PARN by two siRNA sequences, and no effects on the expression of CYRANO RNA. **i,** Northern blot of endogenous miR-7-5p and miR-21-5p on wild-type and DIS3L2 KO cells upon the treatment with control siRNA, siEXOSC3 or siPARN, with or without ectopic expression of CYRANO plasmid. **j,** Quantitating the Northern blot density of the miR-7-5p on wild-type cells upon the treatment with control siRNA, siEXOSC3 or siPARN. p-values were calculated using two-tailed paired t-test. (N=3 independent experiments). Source data are provided as a Source Data file.

Supplementary Table 1

	**NORTHERN BLOTTING PROBES**
hsa-mir-1-3p probe	GATACATACTTCTTTACATTCCA
hsa-miR-122-5p probe	GCAAACACCATTGTCACACTCCA
hsa-miR-15a-5p probe	GCACAAACCATTATGTGCTGCTA
hsa-miR-27a-5p probe	GTGCTCACAAGCAGCTAAGCCCT
hsa-miR-27a-3p probe	GCGGAACTTAGCCACTGTGAA
hsa-miR-27a-loop probe	GTGAACACGACTTGGTGTGGA
hsa-miR-27a mimic-5p probe	GTTCACAGTGGCTAAGTTCCGC
hsa-miR-23a-5P probe	GAAATCCCATCCCCAGGAACCCC
hsa-miR-23a-3p probe	GGAAATCCCTGGCAATGTGAT
hsa-miR-10b-5p probe	GAAATTCGGTTCTACAGGGTA
hsa-miR-7-5p probe	GACAAAATCACTAGTCTTCCA
hsa-miR-148a-3p probe	GACAAAGTTCTGTAGTGCACTGA
hsa-miR-222-3p probe	GACCCAGTAGCCAGATGTAGCT
hsa-miR-769-5p probe	GAGCTCAGAACCCAGAGGTCTCA
hsa-miR-21-5p probe	GTCAACATCAGTCTGATAAGCTA
U6 snRNA probe	GCCATGCTAATCTTCTCTGTAT
Tyr-tRNA probe	TCCGCTCTACCAGCTGAGCTATCGAAGG
	**CLONING PCR PRIMERS**
pri-miR-15a-F	GACAAGCTTGCGGCCGCAGCATTTAGTTGTATTGCCCTGTT
pri-miR-15a-R	ATCCTCTAGAGTCGACTAAGAGATGATACATCTGAAAAATTAG
pri-miR-1-2-F	GACAAGCTTGCGGCCGCATCTTAAGTTTCCGAAATACACTCT
pri-miR-1-2-R	ATCCTCTAGAGTCGACATAATGAGAGTGCCTAATTTAAAAATG
pri-miR-122-F	GACAAGCTTGCGGCCGCAATTGTTGCAAACAGAGTTCCTG
pri-miR-122-R	ATCCTCTAGAGTCGACTGCAATCTTTTTATTGACTTGGGAA
Dis3L2-Infusion-F	CCCGATTACGCTAGCAGCCATCCTGACTACAGAATGAACC
Dis3L2-Infusion-R	GGATCCACTGAATTCTCAGCTGGTGCTTGAGTCCTCGGGC
DIS3L2-mut-F	CCGAGACCTCAATGATGCCCTCT
DIS3L2-mut-R	GCGGTTGATGGGTCAATG
lentiCRISPR v2-puro-DIS3L2-1-F	CACCGTTCTTTGACTTTTTGTCACC
lentiCRISPR v2-puro-DIS3L2-1-R	AAACGGTGACAAAAAGTCAAAGAAC
lentiCRISPR v2-puro-DIS3L2-2-F	CACCGTCCAAGGAGGATGTTTCAGA
lentiCRISPR v2-puro-DIS3L2-2-R	AAACTCTGAAACATCCTCCTTGGAC
lentiCRISPR v2-puro-DIS3L2-3-F	CACCGACGTCGGCAAAGCGGCGGAT
lentiCRISPR v2-puro-DIS3L2-3-R	AAACATCCGCCGCTTTGCCGACGTC
lentiCRISPR v2-puro-DIS3L2-4-F	CACCGCTCCTCGCTGCTATGCTCTG
lentiCRISPR v2-puro-DIS3L2-4-R	AAACCAGAGCATAGCAGCGAGGAGC
lentiCRISPR v2-puro-DIS3L2-5-F	CACCGATGGGGGGCAGCTCTTTCGC
lentiCRISPR v2-puro-DIS3L2-5-R	AAACGCGAAAGAGCTGCCCCCCATC
lentiCRISPR v2-puro-DIS3L2-6-F	CACCGGCCATGGACAATTCCACCAG
lentiCRISPR v2-puro-DIS3L2-6-R	AAACCTGGTGGAATTGTCCATGGCC
lentiCRISPR v2-puro-DIS3L2-7-F	CACCGCTTGTCATCTCCAAATGTTT
lentiCRISPR v2-puro-DIS3L2-7-R	AAACAAACATTTGGAGATGACAAGC
lentiCRISPR v2-puro-DIS3L2-8-F	CACCGACGTCGGCAAAGCGGCGGAT
lentiCRISPR v2-puro-DIS3L2-8-R	AAACATCCGCCGCTTTGCCGACGTC
lentiCRISPR v2-puro-DIS3L2-9-F	CACCGCGCGCTTGGACGCCATGCGG
lentiCRISPR v2-puro-DIS3L2-9-R	AAACCCGCATGGCGTCCAAGCGCGC

lentiCRISPR v2-puro-DIS3L2-10-F	CACCGGCACCAGGACGTCGGCAAAG
lentiCRISPR v2-puro-DIS3L2-10-R	AAACCTTTGCCGACGTCCTGGTGCC
	**siRNA**
CYRANO-siRNA1-F	rGrGrCrUrGrArGrUrUrUrCrArUrUrUrGrArArArCrArGrGTG
CYRANO-siRNA1-R	rCrArCrCrUrGrUrUrUrCrArArArUrGrArArArCrUrCrArGrCrCrUrU
CYRANO-siRNA2-F	rCrArUrGrCrArGrUrGrCrCrArUrCrUrGrArCrUrUrUrArUGG
CYRANO-siRNA2-R	rCrCrArUrArArArGrUrCrArGrArUrGrGrCrArCrUrGrCrArUrGrArG
EXOSC3 siRNA-1	GUAUUAUUAGAGUCCGAAA
EXOSC3 siRNA-2	GGAGUGAGCCAGCUUCUUU
PARN siRNA-1	GAAAAGAAGGAGCGAUAUA
PARN siRNA-2	CAUGAGAGGGCUUGCCGUA
	**REAL-TIME PCR PRIMER**
CYRANO-F1	TGCGAAGATGGCGGAGTAAG
CYRANO-R1	TAGTTCCTCTCCTCTGGCCG
CYRANO-F2	TTCCAGTTTCAGCCACTACCA
CYRANO-R2	TCACAGGATGAGCCAGGATTT
EXOSC3-F	GGAGTGAGCCAGCTTCTTTG
EXOSC3-R	AGATGAGATCTCCAACCTGCAC
PARN-F	GCTCTTGGACGTCATGCACA
PARN-R	AAAAGGTTGTGTGCTGGCCA
GAPDH-F	AACGGGAAGCTTGTCATCAATGGAAA
GAPDH-R	GCATCAGCAGAGGGGGCAGAG
	**GBLOCK**
CYRANO	AATTCTAGGCGATCGCTCGAGGTTAAAACCGGGATATGTGCAATAGAAATATATATATATATATGTATATTTTAATACTTGTGGACAAATGTTACAAGTTGTTTAAGAACAACAAAATCACCAATGTCTTCCATTTTGAGATGTGTATAGTTTTGTAAGCATTAGTGCTTGGTAGCATATTGTAGTGCCATGTTAGGGGTTAGTGCATGAGTCTAGTGACTAGTGTCGACGCGGCCGCT
CYRANO-mut	AATTCTAGGCGATCGCTCGAGGTTAAAACCGGGATATGTGCAATAGAAATATATATATATATATGTATATTTTAATACTTGTGGACAAATGTTACAAGTTGTTTAAGGCGGAACTTAGAAUCACTGTGAATTTTGAGATGTGTATAGTTTTGTAAGCATTAGTGCTTGGTAGCATATTGTAGTGCCATGTTAGGGGTTAGTGCATGAGTCTAGTGACTAGTGTCGACGCGGCCGCT
