# Supplementary material for: AGO-bound mature miRNAs are oligouridylated by TUTs and subsequently degraded by DIS3L2
Source: Nat Commun. 2020 Jun 2;11:2765. doi: 10.1038/s41467-020-16533-w (PMC7265490; doi:10.1038/s41467-020-16533-w)
Supplement: Supplementary file 3 — Reporting Summary [file 41467_2020_16533_MOESM3_ESM.pdf]

nature research

Corresponding author(s): Shuo Gu

Last updated by author(s): Apr 20, 2020

Reporting Summary

Nature Research wishes to improve the reproducibility of the work that we publish. This form provides structure for consistency and transparency in reporting. For further information on Nature Research policies, see Authors & Referees and the Editorial Policy Checklist.

Statistics

For all statistical analyses, confirm that the following items are present in the figure legend, table legend, main text, or Methods section.

n/a	Confirmed	
☐	☒	The exact sample size (*n*) for each experimental group/condition, given as a discrete number and unit of measurement
☐	☒	A statement on whether measurements were taken from distinct samples or whether the same sample was measured repeatedly
☐	☒	The statistical test(s) used AND whether they are one- or two-sided<br>*Only common tests should be described solely by name; describe more complex techniques in the Methods section.*
☒	☐	A description of all covariates tested
☒	☐	A description of any assumptions or corrections, such as tests of normality and adjustment for multiple comparisons
☐	☒	A full description of the statistical parameters including central tendency (e.g. means) or other basic estimates (e.g. regression coefficient) AND variation (e.g. standard deviation) or associated estimates of uncertainty (e.g. confidence intervals)
☐	☒	For null hypothesis testing, the test statistic (e.g. *F*, *t*, *r*) with confidence intervals, effect sizes, degrees of freedom and *P* value noted<br>*Give P values as exact values whenever suitable.*
☒	☐	For Bayesian analysis, information on the choice of priors and Markov chain Monte Carlo settings
☒	☐	For hierarchical and complex designs, identification of the appropriate level for tests and full reporting of outcomes
☒	☐	Estimates of effect sizes (e.g. Cohen's *d*, Pearson's *r*), indicating how they were calculated

Our web collection on statistics for biologists contains articles on many of the points above.

Software and code

Policy information about availability of computer code

Data collection	Genomic Data Commons (TCGA mutations), miRNA-seq TCGA (controlled data, dbGAP protected), miRNA-seq (Illumina Miseq/Hiseq)
Data analysis	Customized scripts (https://github.com/Gu-Lab-RBL-NCI/oligo-tail-miRNA), QuagmiR (Bioinformatics, 2018), Rstudio v1.1.456, Prism v.8

For manuscripts utilizing custom algorithms or software that are central to the research but not yet described in published literature, software must be made available to editors/reviewers. We strongly encourage code deposition in a community repository (e.g. GitHub). See the Nature Research guidelines for submitting code & software for further information.

Data

Policy information about availability of data

All manuscripts must include a data availability statement. This statement should provide the following information, where applicable:

- Accession codes, unique identifiers, or web links for publicly available datasets
- A list of figures that have associated raw data
- A description of any restrictions on data availability

All the figures are included in this article (and its supplementary information files). The NGS datasets generated during and/or analyzed during the current study are available in the GEO with the accession codes GSE139567 and GSE121327. The Source Data file is available at Mendeley (http://dx.doi.org/10.17632/s5hss3jw6k.2)

Field-specific reporting

Please select the one below that is the best fit for your research. If you are not sure, read the appropriate sections before making your selection.

☒ Life sciences ☐ Behavioural & social sciences ☐ Ecological, evolutionary & environmental sciences

Life sciences study design

All studies must disclose on these points even when the disclosure is negative.

Sample size	No sample size calculation was performed. We selected the sample size based on estimated effects and data quality to ensure representative results and sufficient statistical power. Data represent at least three independent experiments each performed in replicates. Single experiments were performed for next-generation sequencing assays where effects are measured across >= 25/50 miRNAs.
Data exclusions	No data were excluded.
Replication	Experiments were replicated successful, and the number of replication were documented in the manuscript.
Randomization	In all cell based experiments control and treated cells were seeded from the same populations and treated simultaneously; the comparison of between wt and KO clones was performed using similar passage of the cells.
Blinding	Data collection and analysis was not performed blindly. All data points were obtained through biochemical or next-generation sequencing assays, with no human involvement on the measurement quantification.

Reporting for specific materials, systems and methods

We require information from authors about some types of materials, experimental systems and methods used in many studies. Here, indicate whether each material, system or method listed is relevant to your study. If you are not sure if a list item applies to your research, read the appropriate section before selecting a response.

Materials & experimental systems

n/a	Involved in the study	
☐	☒	Antibodies
☐	☒	Eukaryotic cell lines
☒	☐	Palaeontology
☒	☐	Animals and other organisms
☒	☐	Human research participants
☒	☐	Clinical data

Methods

n/a	Involved in the study	
☒	☐	ChIP-seq
☒	☐	Flow cytometry
☒	☐	MRI-based neuroimaging

Antibodies

Antibodies used	anti-DIS3L2 (1:1000, Sigma #HPA035797), anti-ZCCHC11 (1:500, Proteintech, #18980-1-AP), anti-ZCCHC6 (1:2000, Proteintech, #25196-1-AP), anti-Tubulin (1:3000, Sigma, #T9026), anti-AGO1 (1:500, Wako, #015-22411), and anti-AGO2 (1:500, Wako, #015-22031)
Validation	Supplementary Fig. 4a validated the specificity of anti-ZCCHC11, anti-ZCCHC6 with KO cells. Supplementary Fig. 4a validated the specificity of anti-DIS3L2 with KO cells. The manufacture's website of anti-Tubulin states "The antibody is specific for α-tubulin in immunoblotting assays and may be used for localization of α-tubulin in cultured cells or tissue sections". The manufacture's website of anti-AGO1 states this antibody cross react to human and mouse AGO1. The manufacture's website of anti-AGO2 states this antibody specific to human AGO2.

Eukaryotic cell lines

Policy information about cell lines

Cell line source(s)	HEK293T is from ATCC. All the KO cell lines are established in the HEK293Tusing CRISPR-Cas9 system.
Authentication	HEK293T is authenticated by Short-tandem repeat profiling.
Mycoplasma contamination	HEK293T from ATCC is tested mycoplasma negtive.
Commonly misidentified lines (See ICLAC register)	none
